# Prognostic factors and survival of patients with uterine sarcoma: a German unicenter analysis

**DOI:** 10.1007/s00404-022-06515-2

**Published:** 2022-07-03

**Authors:** Alexandra Huss, Maximilian Klar, Mir Fuad Hasanov, Ingolf Juhasz-Böss, Michaela Bossart

**Affiliations:** Department of Obstetrics and Gynecology, Faculty of Medicine, University of Freiburg, Hugstetterstr. 55, 79106 Freiburg, Germany

**Keywords:** Uterine sarcoma, Survival analysis, Leiomyosarcoma, Low-grade endometrial stromal sarcoma, High-grade endometrial stromal sarcoma, Prognostic factors

## Abstract

**Purpose:**

Uterine sarcoma (US) as a histologically heterogeneous group of tumors is rare and associated with poor prognosis. Prognostic factors based on systematic data collection need to be identified to optimize patients’ treatment.

**Methods:**

This unicenter, retrospective cohort study includes 57 patients treated at the University Hospital Freiburg, Germany between 1999 and 2017. Progression-free survival (PFS) and overall survival (OS) were calculated and visualized in Kaplan–Meier curves. Prognostic factors were identified using log-rank test and Cox regression.

**Results:**

44 Leiomyosarcoma (LMS), 7 low-grade endometrial stromal sarcoma (LG-ESS), 4 high-grade ESS and 2 undifferentiated US patients were identified. The median age at time of diagnosis was 51.0 years (range 18–83). The median follow-up time was 35 months. PFS for the total cohort was 14.0 (95%-Confidence-Interval (CI) 9.7–18.3) and OS 36.0 months (95%-CI 22.1–49.9). Tumor pathology was prognostically significant for OS with LG-ESS being the most favorable (mean OS 150.3 months). In the multivariate analysis, patients over 52 years showed a four times higher risk for tumor recurrence (hazard ratio (HR) 4.4; 95%-CI 1.5–12.9). Progesterone receptor negativity was associated with a two times higher risk for death (HR 2.8; 95%-CI 1.0–7.5). For LMS patients age ≥ 52 years (*p* = 0.04), clear surgical margins (*p* = 0.01), FIGO stage (*p* = 0.01) and no application of chemotherapy (*p* = 0.02) were statistically significant factors for OS.

**Conclusion:**

Tumor histology, age at time of diagnosis and progesterone receptor status were prognostic factors for US. Unfavorable OS in LMS patients was associated with advanced FIGO stage, suboptimal cytoreduction and application of chemotherapy.

## Introduction

Uterine sarcoma (US) is a very rare mesenchymal tumor entity with an incidence of 3% of all uterine malignancies [1–3]. It appears more frequently in women over 50 years of age.

The World Health Organization (WHO) classification system of 2014 divides US in four subtypes: leiomyosarcoma (LMS), low- and high-grade endometrial stromal sarcoma (LG-ESS and HG-ESS), undifferentiated uterine sarcoma (UUS) and adenosarcoma (AS) [4]. LMS is the most common with 60–70%, low and high-grade ESS as well as UUS are each diagnosed in 10%, AS and heterologous sarcomas in 5% [5]. Prognosis of US is poor with 70% tumor recurrence and 5-year overall survival (OS) rates around 40% [5, 6]. Diagnosis of this tumor entity is generally late due to unspecific early symptoms, resembling uterine myoma or adenomyosis. In more than half of all cases, the finding of US is incidental [7]. Neither curettage nor preoperative imaging are able to securely exclude US [5, 8]. Primarily treatment is surgical, including hysterectomy without morcellation together with bilateral adnexectomy and in advanced stages complete cytoreduction. Systematic lymphadenectomy is not mandatory. So far, no adjuvant therapy could show convincing benefit on patients’ survival. For LG-ESS with positive hormonal receptor status adjuvant endocrine therapy is offered. Close postoperative follow-up care, consisting of gynecologic examination and sonography, is mandatory. The histological workup and diagnosis are essential, but very often challenging. Frequently, immunohistochemically or additional molecular pathological methods as well as a reference pathological opinion are needed [9].

The aim of this study was to provide survival data and identify prognostic factors of patients with US from a German tertiary academic hospital.

## Methods

### Patient cohort

All patients treated for US at the Department of Obstetrics and Gynecology at University of Freiburg (UFK), Germany, between June 1999 and August 2017 over 18 years of age were included into this study.

### Methods

This study was conducted retrospectively based on our clinical and pathology data. General patient characteristics were age and menstrual status at time of diagnosis. Tumor stage was defined according to the 2014 International Federation of Gynecology and Obstetrics (FIGO) classification, surgical margins (R0, R1, Rx) and grading were included. Histopathological workup was analyzed collecting histological type of uterine sarcoma, hormone receptors (estrogen receptor, progesterone receptor (PR), > 5% defined as positive) and proliferation index. Adjuvant treatment strategies, including radiotherapy, chemotherapy and endocrine therapy were specified.

Progression-free survival (PFS) and overall survival (OS) were calculated as time in months from date of diagnosis to date of recurrence, respectively, to date of last follow-up or date of death.

SPSS 20.0 was used for all statistical workup: OS and PFS were visualized using Kaplan–Meier curves. For the calculation of prognostic factors for the total cohort and for LMS tumors for PFS and OS, log-rank tests were performed for univariate analysis and the Cox-proportional hazards regression models for multivariate analyses. The variables were histological tumor type, age at time of diagnosis, menopausal status, surgical margin, FIGO stage, PR status and use of chemotherapy. *p* value < 0.05 was considered to indicate differences of statistical significance.

The study was approved by the ethics committee of University Hospital Freiburg (application number 349/19) meeting all institutional guidelines. Patient consent was requested during initial hospitalization and due to pseudonymized, retrospective study design no additional patient consent was needed.

## Results

57 patients with US treated between June 1999 and August 2017 with last follow-up in March 2020 were included into this study. The observation period was 250 months and the median time of follow-up was 35 months (range 4–240 months).

### Descriptive analysis

#### Descriptive analysis of the total cohort

At time of first diagnosis the patients were between 18 and 83 years of age. Based on the median age at first diagnosis, 51 years was used as an age cut-off. Group one (‘young age’) was defined as patients between 18 and 51 years at time of first diagnosis, group two with patients older than 52 years (‘old age’). The most common histopathological subtype was LMS with 44 cases. ESS was found in 11 patients, UUS was diagnosed in two patients. Patients’ characteristics are presented in Table 1.

**Table 1:** Clinical and histopathological characteristics of the total cohort and of LMS patients

Characteristics total cohort			Characteristics of LMS patients number percentage	
	Number	Percentage		
Age at diagnosis	51 years ± 12.2		51 years ± 11.4	
Histological subtype				
LMS	44	77.2		
LG-ESS	7	12.3		
HG-ESS	4	7.0		
UUS	2	3.5		
Menstrual status				
Premenopausal	26	45.6	21	47.7
Postmenopausal	26	45.6	18	40.9
Missing data	5	8.8	5	11.4
FIGO stage				
Stage I	36	63.2	30	68.2
Stage II	7	12.3	3	6.8
Stage III	3	5.3	3	6.8
Stage IV	11	19.3	7	15.9
Missing data	1	1.8	1	2.3
Tumor grading				
Grade 1	11	19.3	4	9.1
Grade 2	13	22.8	13	29.5
Grade 3	19	33.3	13	29.5
Missing data	14	24.6	14	31.8
Progesterone receptor status				
PR +	20	35.1	17	38.6
PR−	16	28.1	6	13.6
Missing data	21	36.8	21	47.7

*LMS* Leiomyosarcoma, *LG-ESS* low-grade endometrial stromal sarcoma, *HG-ESS* high-grade ESS, *UUS* undifferentiated uterine sarcoma, *FIGO* International Federation of Gynecology and Obstetrics, *PR* progesterone receptor

All patients received primary surgical treatment. R0 is defined as no microscopic residual tumor. In advanced cases, R0 means optimal surgical treatment and is defined as locally no microscopic residual tumor in the pathology report and no macroscopical residual tumor according to the surgical report. R0 was reached in 63.16% of cases. In ten cases, there was at least microscopical tumor left (R1). Rx was found in 11 cases, including 7 cases with morcellation (six LMS, one LG-ESS). Four patients were initially treated externally. Three patients were treated with primary laparoscopic operation at UFK with morcellement due to preoperatively benign assumption, all of them received a following surgery immediately after diagnosis of malignancy. No residual tumor could be found in the second operation macroscopically and microscopically. Patients with morcellation treatment had a mean age at first diagnosis of 43.7 years.

50.9% of patients underwent any kind of adjuvant therapy. In 24 cases (42.1%), the patient received chemotherapy at any point through course of disease. Most patients were treated with Docetaxel/Gemcitabine (13 cases) for first-line treatment at recurrent disease. Other patients received Ifosfamid, partially in combination with Etoposid. In further lines, Dacarbazin, Doxorubicin, Adriamycin and Platin were used.

#### Descriptive analysis of sub cohorts stratified by histopathological type

The subgroup LMS includes 44 patients with a median age at time of diagnosis of 51 years. The majority presented with a tumor confined to the uterus (Table 1). 16 patients in FIGO stage I, 1 in stage II and III and 3 patients in FIGO stage IV received no adjuvant treatment. 16 patients received adjuvant treatment, in early FIGO stages, mostly radiation and endocrine therapy. In advanced stages chemotherapy was used, in two cases in combination with radiation. 21 patients received chemotherapy at any point of time through course of disease. Four patients (9.1%) survived the trial observation period recurrence-free. 75% of LMS patients were reported dead, seven patients survived with relapse (15.9%).

LG-ESS was diagnosed in seven patients with a mean age at time of diagnosis of 48 years. Four patients received adjuvant endocrine treatment, two in FIGO stage I, one in stage II and one in stage IV. Five patients were still alive at the end of the observation period, two without tumor relapse.

Four patients were diagnosed with a HG-ESS. One patient was diagnosed in FIGO stage I and has no recurrence after 34 months. Two patients presented in FIGO stage IV. In the other three cases, the tumor recurred after 4, 5 and 23 months, respectively.

### Survival analysis

Median time of follow-up among all subgroups was 35 months (4–240 months). At the end of the observation period, 39 patients (66.1%) were reported dead. At the end of the observation period, eight patients (14.0%) survived recurrence-free, ten patients were alive with tumor recurrence.

**Fig. 1:**
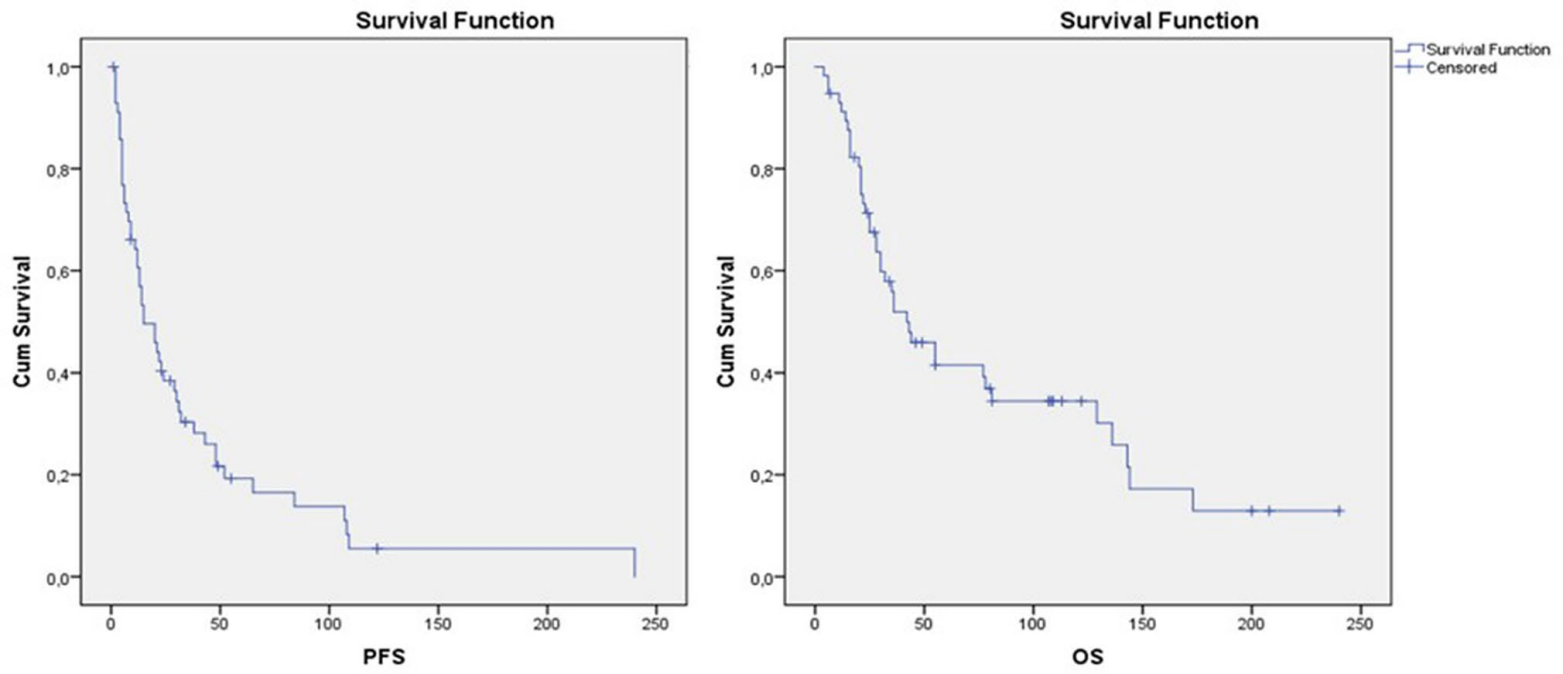
Kaplan–Meier curves for progression-free survival (PFS) and overall survival (OS) of the total cohort

Median progression-free survival (PFS) for the whole cohort was 15.0 months (95% CI 6.8–23.2). Median overall survival (OS) was 42.0 months (95% CI 23.21–60.79), visualized in Kaplan–Meier curves in Fig. 1. The 5-year OS rate was 33.0% ± 7.0%.

### Prognostic factors

#### Univariate analysis of the total cohort

Univariate analysis was performed using logistic regression by log-rank test. For further analyzes, cases were divided in three groups according to FIGO stage, pairing FIGO II and III in one group due to the small number of cases.

Stratified by histopathological subgroup (Table 2), LMS showed a median PFS of 15 months and an OS of 43 months. LG-ESS showed a longer PFS and OS with a 5-year OS rate of 69.0% ± 18.0%. HG-ESS patients had a median PFS of 5 months and OS of 6 months. Both patients with UUS died during observation period (after 7 and 30 months). Pathological subtype was a significant prognostic marker for OS (*p* 0.04).

**Table 2:** Progression-free survival (PFS) and overall survival (OS) stratified by histopathological subtype

Histopathological subtype	PFS [months] (95% CI)	OS [months] (95% CI)	5-year OS Rate
LMS	15 (7.7–22.3)	43 (25.7–60.3)	29.0% ± 8.0%
LG-ESS	38 (9.3–66.7)	150 (83.4–217.1)	69.0% ± 18.0%
HG-ESS	5 (0–23.6)	6 (0–29.5)	

95% Confidence Interval

*LMS* Leiomyosarcoma, *LG-ESS* low-grade endometrial stromal sarcoma, *HG-ESS* high-grade ESS, *UUS* undifferentiated uterine sarcoma

Patients diagnosed in FIGO stage I had the longest PFS and OS (Table 3).

**Table 3:** Univariate analysis: median progression-free survival (PFS) and overall survival (OS) in months stratified by FIGO stage, age, application of chemotherapy and progesterone receptor status

	Median PFS total cohort (95% CI)	*p* value	Median OS total cohort (95% CI)	*p* value	Median PFS LMS patients (95% CI)	*p* value	Median OS LMS patients (95% CI)	*p* value
FIGO I	20 (6.8–33.2)	0.6	55 (11.6–98.4)	0.35	15 (5.3–24.7)	0.18	55 (1.8–108.2)	0.01
FIGO II	6 (3.4–8.6)		30 (11.5–48.5)		5 (2.6–7.4)		21 (12.4–29.6)	
FIGO III	14 (0.0–30.0)		25 (10.6–39.4)					
FIGO IV	15 (12.1–17.9)		23 (9.1– 36.9)		15 (0.0–35.4)		23 (5.0–40.9)	
‘young age’ 18–51 years	23 (7.7–38.3)	0.01	81.0 (15.5–146.5)	0.003	20.0 (7.5–32.5)	0.08	78 (25.2–130.8)	0.04
‘old age’ ≥ 52 years	9 (0.3–17.8)		32.0 (21.5–42.5)		9.0 (0.0–22.1)		35 (23.9–46.1)	
Chemotherapy	8 (0.3–15.7)		28 (14.8–41.2)		8 (0.0–17)		30 (13.6–46.4)	
No chemotherapy	30 (8.3–51.7)	0.001	136 (25.9–246.1)	0.015	29 (0.0–60.2)	0.007	136 (36.2–235.8)	0.02
Tumor progesterone positive	22 (15.4–28.6)	0.026	136 (15.9–256.1)	0.03	15 (4.2–25.8)	0.12	136 (20.8–251.2)	0.4
Tumor progesterone negative	9 (0–21.8)		30 (12.3–47.7)		5 (0.0–15.8)		36 (3.6–68.4)	
Surgical margin R0	13 (0.0–26.2)	0.5	44 (19.6–68.3)	0.13	13 (0.0–26.0)	0.6	55 (34.0–76.0)	0.008
R1	15 (0.9–29.1)		23 (9.1–36.9)		15 (0.0–43.3)		16 (8.4–23.6)	
Rx	23 (6.8–23.2)		143 (0.0–290.3)		14 (9.8–18.2)		143 (n.a.)	

LMS leiomyosarcoma, *95% CI* 95% confidence Interval, *FIGO* International Federation of Gynecology and Obstetrics, *N.a.* not achieved

Differences in surgical margins were not statistically significant. Rx includes seven cases with morcellation treatment. In three cases after morcellation, no residual tumor was found after subsequent open hysterectomy. In one case, the tumor recurred after 29 months, all three patients are still alive at the end of the observatin period with an OS of 29 months.

**Fig. 2:**
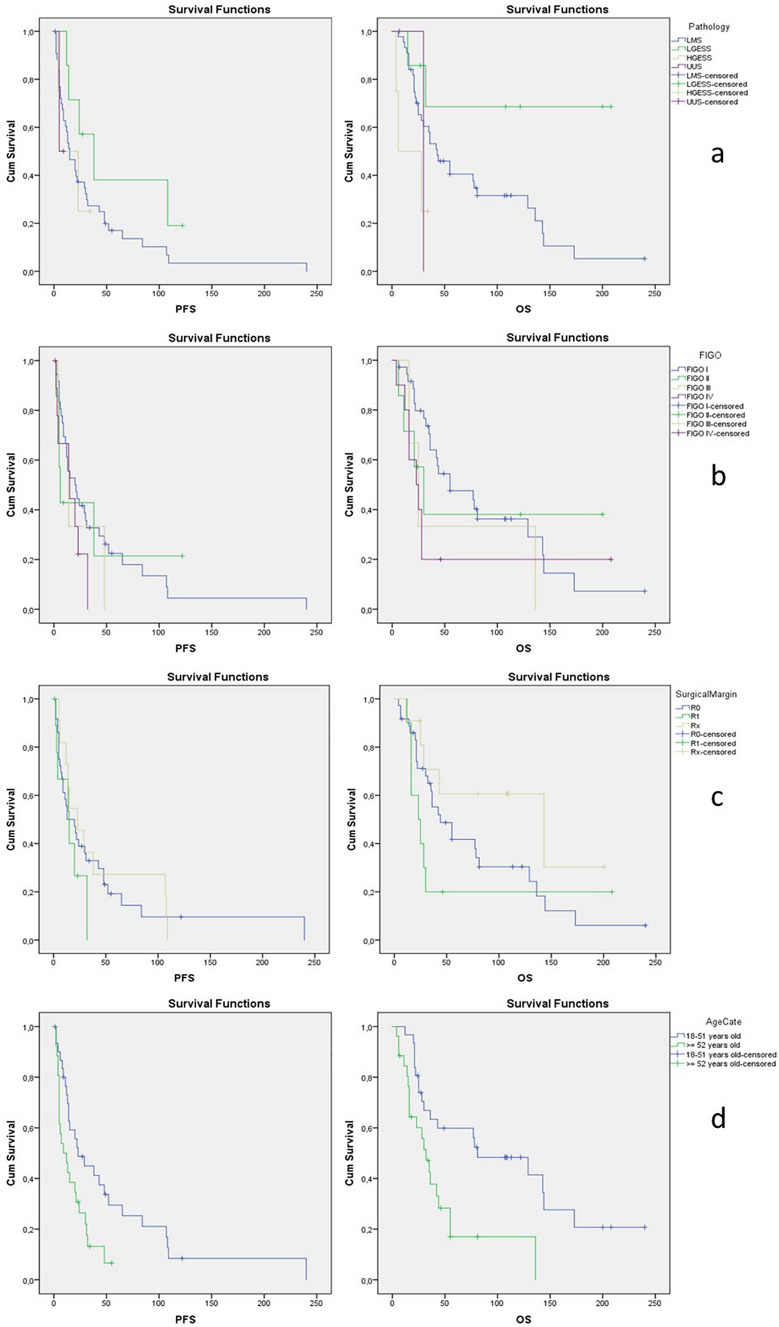
Kaplan–Meier curves for progression-free survival (PFS) and overall survival (OS) stratified by a: pathological subtype, b: FIGO stage, c: surgical margin and d: age category at time of diagnosis over or under 52 years

‘Old age’ was a statistically significant negative prognostic factor for PFS (p 0.01) and for OS (*p* 0.003). Application of chemotherapy throughout course of disease was a statistically negative prognostic factor for median PFS and median OS (136 vs 28 months, *p* 0.015). US patients had a significantly longer PFS and OS when the tumor was PR positive. Results of univariate analysis are shown in Table 3 and visualized in Fig. 2.

#### Multivariate analysis of the total cohort

The results of the multivariate analysis are shown in Table 4. ‘Young age’ and R0 resection of the tumor were significant positive prognostic factors for PFS. Patients with complete tumor resection had a five times better PFS than people with R1 resection. PR status positive was the only significant positive prognostic factor for OS (*p* 0.049).

**Table 4:** Multivariate analysis for median progression-free survival (PFS) and overall survival (OS) for the total cohort and for the sub cohort Leiomyosarcoma (LMS)

	PFS total cohort		OS total cohort		PFS LMS patients		OS LMS patients	
	HR (95% CI)	*p*-value	HR (95% CI)	*p*-value	HR (95% CI)	*p*-value	HR (95% CI)	*p*-value
Progesterone receptor positivity	2.25 (1.0–5.1)	0.05	2.75 (1.0–7.5)	0.049	1.96 (0.6–3.0)	0.24	1.97 (0.5–7.2)	0.3
‘Young age ‘ vs ‘old age ‘	4.43 (1.5–12.9)	0.006	3.04 (0.9–9.9)	0.07	1.41 (0.4–4.9)	0.59	1.46 (0.3–7.5)	0.65
Surgical margin R0 vs R1	5.36 (1.2–24.9)	0.03	0.82 (0.1–5.0)	0.83	15.27 (0.8–302.4)	0.07	11.19 (0.6–197.7)	0.1
Surgical margin R0 vs Rx	2.35 (0.7–7.6)	0.16	1.07 (0.3–4.9)	0.93	1.24 (0.3–4.6)	0.75	1.05 (0.2–5.8)	0.96
FIGO stage I vs II/III	0.99 (0.4–8.7)	0.98	2.02 (0.6–7.1)	0.27	1.36 (0.4–5.4)	0.66	1.72 (0.3–10.6)	0.56
FIGO stage I vs IV	1.89 (0.5–6.9)	0.33	3.24 (0.5–19.9)	0.21	2.16 (0.1–49.9)	0.63	0.55 (0.0–11.9)	0.7

*HR* hazard ratio, *95% CI* 95% confidence interval, *FIGO* International Federation of Gynecology and Obstetrics

#### Prognostic factors in LMS tumors

In the univariate analysis, age ≥ 52 years (*p* 0.04), surgical margin (*p* 0.01), FIGO stage (*p* 0.01) and no application of chemotherapy (*p* 0.02) were statistically significant factors for OS in LMS patients. PR status was not statistically significant (Table 3).

In the multivariate analysis, no variable was significantly prognostic. PR status was a significant prognostic factor for the total cohort for PFS and the only significant factor for OS. It was not significant in the LMS patient cohort analyzes, but a trend could be shown.

Prognostic factors for LG-ESS, HG-ESS and US have not been further analyzed due to the small case numbers. In LG-ESS tumors, the four patients with endocrine therapy showed a mean OS time of 373.5 months and were all alive at last follow-up.

## Discussion

This is a retrospective unicenter study on 57 uterine sarcoma patients with an inclusion period of 18 years and a median follow-up of 35 months.

Age over 52 years was a negative prognostic factor for PFS and OS in the univariate analysis of the total cohort, as well as for PFS in the multivariate analysis. For LMS patients, it was statistically significant in the univariate analyses for OS. This accords to prior findings in LMS patients in the SEER database (patients under or 52 years: disease-specific survival 73.5% vs over 52 years 56.1%, *p* < 0.001) [10].

Preoperative diagnosis is often challenging and sometimes leads to inadequate surgical treatment. In this report, the initial malignant diagnose LMS was missed in three cases and was only diagnosed at tumor recurrence. The risk of the occurrence of unexpected LMS during hysterectomy or myomectomy for presumed benign fibroids according to the American Food and Drug Administration is 1 in 1100 women undergoing surgery [11]. According to a Dutch study, women between 40 and 50 years presenting with abnormal uterine bleeding are most at risk for unexpected LMS [12]. This aligns with the fact that patients with morcellation treatment in this study had a mean age at first diagnosis of 43.7 years. Morcellation is an iatrogenic, negative prognostic factor for recurrence with a threefold increase in PFS [13, 14]. An impact on OS could not consistently be shown by reviews [14–17]. Three cases with initial morcellation of the sarcoma were included in our study. Subsequent staging laparotomy and hysterectomy revealed no residual tumor followed by a long OS with 99 months and all three patients still alive. These data are in contrast to other published data and might point out isolated cases in a small cohort but suggests to further investigate the value of second look laparotomy and hysterectomy.

Known prognostic factors on PFS and OS in LMS patients are tumor stage as well as tumor size, age, the amount of mitotic figures, a complete surgical resection and blood vessel invasion [5, 10, 18–21]. To get a useful sample size in rare tumors at one center, long observation periods are needed [22]. Change of classifications as well as modification in histopathological workup over time impede retrospective analyses. In our analysis neither for the total cohort, nor for LMS patients, FIGO stage could show a significant impact on PFS or OS. PR negativity was a negative prognostic marker in our univariate and multivariate analyses for uterine sarcomas. For LMS in univariate analyses, it was a significant negative variable for both PFS and OS. This was confirmed for PFS but not for OS in multivariate analysis, though there was a trend to shorter OS. In a small 25 case study, PR as well as androgen receptor expression was associated with longer disease free survival (DFS) but did not correlate with OS [23]. In a Norwegian study, higher PR score was related to longer OS in a series of 147 stage I LMS tumors [24]. Multicenter databases with larger sample size are warranted for further investigation.

Survival benefit of adjuvant treatment of LMS was not yet shown in randomized controlled trials [5]. Following German guidelines, radiation should not be performed in stage I and II tumors after complete surgical resection [8, 25]. In these stages, adjuvant chemotherapy can be discussed with positive effects on PFS and OS incorporating toxicities based on a study with 23% carcinosarcoma patients [26, 27]. In a study by Ricci et al., neither chemotherapy nor radiation was able to lower the recurrence rate of LMS, but chemotherapy did have an impact on overall survival in stage I and II LMS patients [28]. In this study, the use of chemotherapy at any point in time during course of disease had a significant negative impact both on PFS and on OS. This could be seen for the total US cohort, as well as for LMS patients. The negative effect could not be confirmed in the multivariate analysis, therefore, chemotherapy could be a confounding factor. Given the known and strong side-effects and toxicities limiting patients’ quality of life, chemotherapy should only be administered after comprehensive patients counseling and joint decision, taking life quality and quantity into account. Further and bigger studies are needed for thorough elaboration. It has been shown that sarcoma patients benefit from multidisciplinary tumor boards at specialized treatment centers including secondary pathologic review prior to treatment [29]. Blay et al. showed that patients had longer recurrence-free survival rates and a trend to longer OS when presented and discussed in a board [29]. This highlights the need of centralized diagnostic and treatment with expert pathological departments.

Since uterine sarcomas and their recurrences are rare cases with probably unfavorable prognosis, treatment tends to individualized strategies. Consequent multicenter registration of every individual case and trial participation would be helpful to build a bigger data pool and provide treatment recommendations for the future. Such is the German registry for gynecologic sarcomas (REGSA) [7, 30]. Testing the use of targeted therapies, for example growth-factor-antibodies, in US is still ongoing [31–33]. International cooperations as the ENGOT rare cancer group are necessary to establish international data collection and launch multinational trials on targeted therapies in rare cancers.

In this study, tumor histology, age at time of diagnosis and progesterone receptor status are significant prognostic markers in univariate analysis. Consequent and standardized immunohistopathological workup as a basis for molecular tumor boards in centralized oncological centers is worthwhile. More randomized controlled trials on adjuvant therapy are necessary to give physicians convincing treatment options especially in the recurrent situation.

## References

[1] Stiller CA, Trama A, Serraino D (2013). Descriptive epidemiology of sarcomas in Europe: Report from the RARECARE project. Eur J Cancer.

[2] D’Angelo E, Prat J (2010). Uterine sarcomas: a review. Gynecol Oncol.

[3] Hosh M, Antar S, Nazzal A, Warda M, Gibreel A, Refky B (2016). Uterine sarcoma: analysis of 13,089 cases based on surveillance, epidemiology, and end results database. Int J Gynecol Cancer Off J Int Gynecol Cancer Soc.

[4] Benson C, Miah AB (2017). Uterine sarcoma—current perspectives. Int J Womens Health.

[5] Denschlag D, Ackermann S, Battista MJ (2019). Sarcoma of the Uterus. Guideline of the DGGG and OEGGG (S2k Level, AWMF Register Number 015/074, February 2019). Geburtshilfe Frauenheilkd.

[6] Survival Rates for Uterine Sarcoma (2021) https://www.cancer.org/cancer/uterine-sarcoma/detection-diagnosis-staging/survival-rates.html. Accessed 7 Mar 2021

[7] Eckes L, Harter P, Muallem MZ, et al (2017) First results of the German prospective Registry for Gynaecological Sarcoma (REGSA). Infobrief NOGGO, 11.12.2017

[8] Juhasz-Böss I, Gabriel L, Bohle RM, Horn LC, Solomayer E-F, Breitbach G-P (2018). Uterine leiomyosarcoma. Oncol Res Treat.

[9] Lopez-Beltran A, Canas-Marques R, Cheng L, Montironi R (2019). Histopathologic challenges: The second OPINION issue. Eur J Surg Oncol.

[10] Kapp DS, Shin JY, Chan JK (2008). Prognostic factors and survival in 1396 patients with uterine leiomyosarcomas. Cancer.

[11] Siedhoff MT, Doll KM, Clarke-Pearson DL, Rutstein SE (2017). Laparoscopic hysterectomy with morcellation vs abdominal hysterectomy for presumed fibroids: an updated decision analysis following the 2014 Food and Drug Administration safety communications. Am J Obstet Gynecol.

[12] van den Haak L, de Kroon CD, Warmerdam MI (2019). Incidence and groups at risk for unexpected uterine leiomyosarcoma: a Dutch nationwide cohort study. Arch Gynecol Obstet.

[13] George S, Barysauskas C, Serrano C (2014). Retrospective cohort study evaluating the impact of intraperitoneal morcellation on outcomes of localized uterine leiomyosarcoma. Cancer.

[14] Ebner F, Wiedenmann S, Bekes I, Wolfgang J, de Gregorio N, de Gregorio A (2019). Results of an internal audit on the survival of patients with uterine sarcoma. J Turk Ger Gynecol Assoc.

[15] Raine-Bennett T, Tucker L-Y, Zaritsky E (2016). Occult uterine sarcoma and leiomyosarcoma: incidence of and survival associated with morcellation. Obstet Gynecol.

[16] Pritts EA, Parker WH, Brown J, Olive DL (2015). Outcome of occult uterine leiomyosarcoma after surgery for presumed uterine fibroids: a systematic review. J Minim Invasive Gynecol.

[17] Ricci S, Stone RL, Fader AN (2017). Uterine leiomyosarcoma: Epidemiology, contemporary treatment strategies and the impact of uterine morcellation. Gynecol Oncol.

[18] Pelmus M, Penault-Llorca F, Guillou L (2009). Prognostic factors in early-stage leiomyosarcoma of the uterus. Int J Gynecol Cancer Off J Int Gynecol Cancer Soc.

[19] Naaman Y, Shveiky D, Ben-Shachar I, Shushan A, Mejia-Gomez J, Benshushan A (2011). Uterine sarcoma: prognostic factors and treatment evaluation. Isr Med Assoc J IMAJ.

[20] Kyriazoglou A, Liontos M, Ziogas DC (2018). Management of uterine sarcomas and prognostic indicators: real world data from a single-institution. BMC Cancer.

[21] Mbatani N, Olawaiye AB, Prat J (2018). Uterine sarcomas. Int J Gynecol Obstet.

[22] Bayar MA, Le Teuff G, Michiels S, Sargent DJ, Le Deley M-C (2016). New insights into the evaluation of randomized controlled trials for rare diseases over a long-term research horizon: a simulation study. Stat Med.

[23] Leitao MM, Soslow RA, Nonaka D (2004). Tissue microarray immunohistochemical expression of estrogen, progesterone, and androgen receptors in uterine leiomyomata and leiomyosarcoma. Cancer.

[24] Davidson B, Kjæreng ML, Førsund M, Danielsen HE, Kristensen GB, Abeler VM (2016). Progesterone receptor expression is an independent prognosticator in FIGO stage I uterine leiomyosarcoma. Am J Clin Pathol.

[25] Reed NS, Mangioni C, Malmström H (2008). Phase III randomised study to evaluate the role of adjuvant pelvic radiotherapy in the treatment of uterine sarcomas stages I and II: an European Organisation for Research and Treatment of Cancer Gynaecological Cancer Group Study (protocol 55874). Eur J Cancer Oxf Engl 1990.

[26] Hensley ML, Wathen JK, Maki RG (2013). Adjuvant therapy for high-grade, uterus-limited leiomyosarcoma: results of a phase 2 trial (SARC 005). Cancer.

[27] Pautier P, Floquet A, Gladieff L (2013). A randomized clinical trial of adjuvant chemotherapy with doxorubicin, ifosfamide, and cisplatin followed by radiotherapy versus radiotherapy alone in patients with localized uterine sarcomas (SARCGYN study). A study of the French Sarcoma Group. Ann Oncol Off J Eur Soc Med Oncol..

[28] Ricci S, Giuntoli RL, Eisenhauer E (2013). Does adjuvant chemotherapy improve survival for women with early-stage uterine leiomyosarcoma?. Gynecol Oncol.

[29] Blay J-Y, Soibinet P, Penel N (2017). Improved survival using specialized multidisciplinary board in sarcoma patients. Ann Oncol.

[30] Armbrust R, Zocholl D, Gimpel A-S, et al (2020) Studienregister—details. https://frauenklinik.uk-koeln.de/forschung/studienregister/studienregister-details/studienregister/regsa/. Accessed 3 Apr 2020

[31] Tap WD, Jones RL, Van Tine BA (2016). Olaratumab and doxorubicin versus doxorubicin alone for treatment of soft-tissue sarcoma: an open-label phase 1b and randomised phase 2 trial. Lancet Lond Engl.

[32] Tap WD, Wagner AJ, Papai Z (2019). ANNOUNCE: A randomized, placebo (PBO)-controlled, double-blind, phase (Ph) III trial of doxorubicin (dox) + olaratumab versus dox + PBO in patients (pts) with advanced soft tissue sarcomas (STS). J Clin Oncol.

[33] Lilly E (2020) A Study of Olaratumab (LY3012207) in Participants With Advanced Soft Tissue Sarcoma - Full Text View - ClinicalTrials.gov. https://clinicaltrials.gov/ct2/show/NCT02659020. Accessed 3 Apr 2020

